# Pancreatic β-cell glutaminase 2 maintains glucose homeostasis under the condition of hyperglycaemia

**DOI:** 10.1038/s41598-023-34336-z

**Published:** 2023-05-05

**Authors:** Hanna Deguchi-Horiuchi, Sawako Suzuki, Eun Young Lee, Takashi Miki, Noriko Yamanaka, Ichiro Manabe, Tomoaki Tanaka, Koutaro Yokote

**Affiliations:** 1 Department of Endocrinology, Hematology and Gerontology, Graduate School of Medicine, Chiba University, Chiba, Japan; 2 Department of Diabetes, Metabolism and Endocrinology, Chiba University hospital, Chiba, Japan; 3 Department of Medical Physiology, Graduate School of Medicine, Chiba University, Chiba, Japan; 4 Department of Disease Biology and Molecular Medicine, Graduate School of Medicine, Chiba University, Chiba, Japan; 5 Department of Molecular Diagnosis, Graduate School of Medicine, Chiba University, Chiba, Japan

**Keywords:** Endocrinology, Energy science and technology

## Abstract

Glutaminase 2 (GLS2), a master regulator of glutaminolysis that is induced by p53 and converts glutamine to glutamate, is abundant in the liver but also exists in pancreatic β-cells. However, the roles of GLS2 in islets associated with glucose metabolism are unknown, presenting a critical issue. To investigate the roles of GLS2 in pancreatic β-cells *in vivo*, we generated β-cell-specific *Gls2* conditional knockout mice (*Gls2* CKO), examined their glucose homeostasis, and validated the findings using a human islet single-cell analysis database. GLS2 expression markedly increased along with p53 in β-cells from control (RIP-Cre) mice fed a high-fat diet. Furthermore, *Gls2* CKO exhibited significant diabetes mellitus with gluconeogenesis and insulin resistance when fed a high-fat diet. Despite marked hyperglycaemia, impaired insulin secretion and paradoxical glucagon elevation were observed in high-fat diet-fed *Gls2* CKO mice. GLS2 silencing in the pancreatic β-cell line MIN6 revealed downregulation of insulin secretion and intracellular ATP levels, which were closely related to glucose-stimulated insulin secretion. Additionally, analysis of single-cell RNA-sequencing data from human pancreatic islet cells also revealed that *GLS2* expression was elevated in β-cells from diabetic donors compared to nondiabetic donors. Consistent with the results of *Gls2* CKO, downregulated GLS2 expression in human pancreatic β-cells from diabetic donors was associated with significantly lower *insulin* gene expression as well as lower expression of members of the insulin secretion pathway, including ATPase and several molecules that signal to insulin secretory granules, in β-cells but higher *glucagon* gene expression in α-cells. Although the exact mechanism by which β-cell-specific GLS2 regulates insulin and glucagon requires further study, our data indicate that GLS2 in pancreatic β-cells maintains glucose homeostasis under the condition of hyperglycaemia.

## Introduction

Glycolysis and glutaminolysis are both essential energy sources. Glutaminase 2 (GLS2) is the critical enzyme that converts glutamine to glutamate in glutaminolysis^1^. We and Hu W. *et al*. identified GLS2 as a p53 target gene^1,2^ and reported that GLS2 promotes aerobic energy production while exerting antioxidant effects *in vitro*^1–3^. Recently, Miller *et al.* have reported that mice with global reduced levels of GLS2 showed lower glucagon-stimulated glutamine-to-glucose flux *in vivo*, which was presumed to be the effect of GLS2 mainly in the liver^4^. Glucose homeostasis is maintained by an intricate and elaborate interaction between the liver, pancreatic β-cells (insulin), pancreatic α-cells (glucagon), and associated organs (e.g., intestines, skeletal muscle, adipose tissue)^5^. GLS2 also exists in pancreatic β-cells^6,7^, and its roles remain incompletely understood. Since the GLS2 metabolite of glutamate is known to involve in multistep insulin secretion, such as proinsulin to insulin conversion and glucose-stimulated insulin secretion in β-cells^8–12^, we hypothesize that GLS2 may also play essential roles in pancreatic islets. To investigate the roles of GLS2 in pancreatic β-cells *in vivo*, we generated β-cell-specific *Gls2* conditional knockout mice (*Gls2* CKO), examined their glucose homeostasis and validated the findings using a human islet single-cell analysis database.

## Results

### β-Cell-specific *Gls2* conditional knockout mice showed significant diabetes mellitus with a paradoxical glucagon increase, impaired insulin secretion and insulin resistance after a high-fat diet

**Figure 1:**
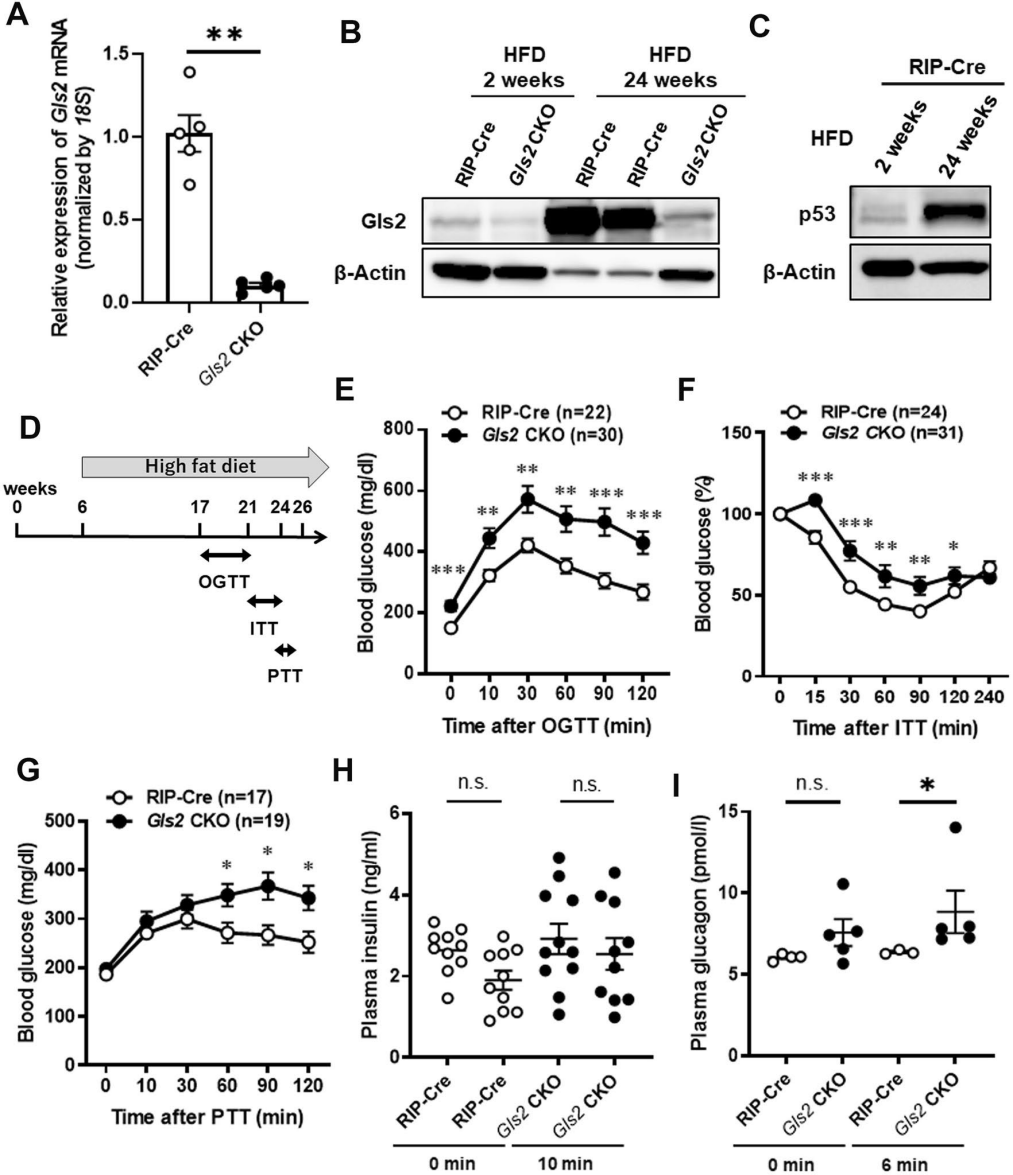
GLS2, p53 and 8-OHdG are upregulated in pancreatic islets from mice fed a high-fat diet, and glucose homeostasis was compared between β-cell-specific *Gls2* conditional knockout mice (*Gls2* CKO) and RIP-Cre mice (control). (**A**), RT–qPCR analysis of *Gls2* in isolated pancreatic islets from *Gls2* CKO and RIP-Cre mice (n=5 each). The expression of *Gls2* RNA was determined by the comparative threshold cycle method and normalized by *18S* expression. Data are shown as the mean ± SEM. **P<0.01. (**B**), Pancreatic islets were isolated from *Gls2* CKO and RIP-Cre mice fed a high-fat diet (HFD) for 2 weeks or 24 weeks followed by immunoblot analysis to detect Gls2 and β-actin. (**C**), Pancreatic islets were isolated from RIP-Cre mice fed a HFD for 2 weeks or 24 weeks followed by immunoblot analysis to detect p53 (FL-393) and β-actin. The blots were cut at 48 kDa before hybridization with p53 or β-actin antibody. (**D**), Timeline of the provocation tests. *Gls2* CKO and RIP-Cre mice fed a HFD were subjected to the indicated provocation tests at 17 to 25 weeks of age. A minimum of 1 week was taken between each loading test. OGTT, oral glucose tolerance test; ITT, Insulin tolerance test; PTT, Pyruvate tolerance test. (**E**–**G**), The blood glucose concentrations during the oral glucose tolerance test (OGTT) at the 17–21 weeks of age *Gls2* CKO and RIP-Cre mice fed a HFD for 11–15 weeks to screen for or diagnose diabetes in graph E. Insulin tolerance test (ITT) at the 21–24 weeks of age *Gls2* CKO and RIP-Cre mice fed a HFD for 15–18 weeks to determine the whole-body sensitivity of insulin receptors by measuring blood glucose level changes before and after insulin administration. The ratio of the change from baseline are shown in F. Pyruvate tolerance test (PTT) at the 22–25 weeks of age *Gls2* CKO and RIP-Cre mice fed a HFD for 16–19 weeks to assess gluconeogenesis in graph G. Data are shown as the mean ± SEM. *P<0.05, **P<0.01, and ***P<0.001 versus RIP-Cre mice. (**H**), Plasma insulin levels at 0 min and 10 min after glucose loading in *Gls2* CKO and RIP-Cre mice (20–23 weeks of age fed a HFD for 14–17 weeks) in E. n.s., not significant. (**I**), Plasma glucagon levels at 0 min and 6 min after glucose loading in *Gls2* CKO and RIP-Cre mice (19–20 weeks of age fed a HFD for 13–14 weeks). *P<0.05. n.s., not significant.

The gene targeting strategy of mice with β-cell-specific *Gls2* exon 2–7 deletion using RIP-Cre transgenic mice is shown in Supplementary Fig. 1. The isolated islets of *Gls2* CKO mice showed a more than 80% reduction in *Gls2* mRNA levels (Fig. 1A) compared to those of RIP-Cre mice. First, an oral glucose tolerance test (OGTT) was performed on 20-week-old *Gls2* CKO and RIP-Cre mice fed chow diets. Although there was no significant difference in body weight between the two groups (Supplementary Fig. 2A), the blood glucose levels were slightly increased in the *Gls2* CKO group (Supplementary Fig. 2B). To further investigate the importance of GLS2 under metabolic stress, mice were challenged with a 60% high-fat diet (HFD) from 6 weeks of age. Notably, the protein levels of Gls2 from isolated pancreatic islets prominently increased 24 weeks after HFD feeding in RIP-Cre mice compared to 2 weeks after HFD, while the levels remained low in *Gls2* CKO mice (Fig. 1B). Similarly, the protein levels of p53 from isolated pancreatic islets (Fig. 1C) and the oxidative stress marker 8-OHdG increased 24 weeks after HFD feeding in RIP-Cre mice (Supplementary Fig. 3A and B). These data indicated that GLS2 increased with p53 induction along with oxidative stress in pancreatic islets after relatively long-term HFD consumption. The loading tests were carried out as shown in Fig. 1D, and *Gls2* CKO mice were compared to RIP-Cre mice. After 11 to 15 weeks on this diet, *Gls2* CKO mice did not present body weight differences (Supplementary Fig. 2C) but showed a significant increase in blood glucose levels at every time point after oral glucose loading compared to levels in RIP-Cre mice (Fig. 1E). Next, the insulin tolerance test (ITT) and pyruvate tolerance test (PTT) were performed for further analysis of insulin resistance and hepatic gluconeogenesis, respectively. The reduction in blood glucose levels or percentage after insulin loading was significantly lower for *Gls2* CKO mice than for RIP-Cre mice, showing insulin resistance (Fig. 1F and Supplementary Fig. 2D). Contrary to the phenomenon that hepatic gluconeogenesis generally decreases during hyperglycaemia, *Gls2* CKO mice revealed a significant increase in blood glucose levels from 60 min after pyruvate administration compared to the levels in RIP-Cre mice, indicating an increase in gluconeogenesis (Fig. 1G). These results suggest that HFD-fed *Gls2* CKO mice show impaired glucose tolerance with an increase in hepatic gluconeogenesis, thereby resulting in insulin resistance. Furthermore, plasma insulin levels did not increase in spite of the high blood glucose in *Gls2* CKO mice (Fig. 1H). Despite significant hyperglycaemia in *Gls2* CKO mice after glucose loading, plasma glucagon, which is generated from pancreatic α-cells and functions as a key regulator of hepatic gluconeogenesis, was increased (Fig. 1I).

### Impairment of Gls2 in pancreatic β-cells resulted in an insulin decrease along with a low intracellular ATP level in β-cells and a paradoxical glucagon increase from α-cells

**Figure 2:**
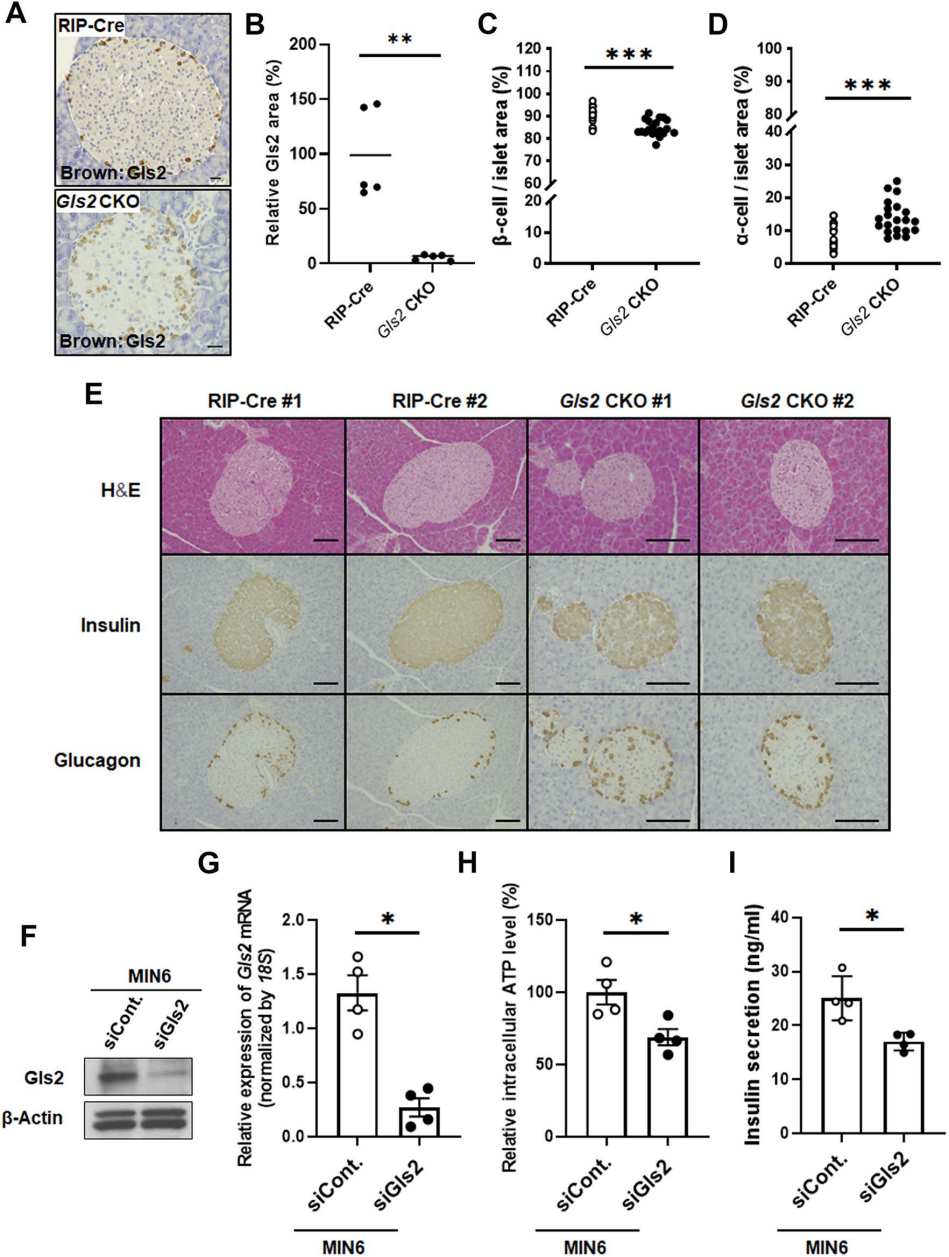
The effect of GLS2 on insulin from β-cells and glucagon from α-cells. (**A**) and (**B**), Immunohistochemical analysis of Gls2 (shown in brown) in pancreas from *Gls2* CKO and RIP-Cre mice. Representative images in A. Scale bars: 20 μm. The relative Gls2 area is shown in dot plots in B. (**C**–**E**), Hematoxylin and eosin (H&E) staining and immunohistochemical analysis of insulin and glucagon in the pancreas from *Gls2* CKO and RIP-Cre mice fed a HFD for 32 weeks were performed. The percentage of insulin-positive β-cells per islet (C) and the percentage of glucagon-positive α-cells per islet (D) were calculated. ***P<0.001. Representative images of the pancreas from HFD-fed *Gls2* CKO and RIP-Cre mice at 32 weeks of age that were stained with hematoxylin and eosin (H&E), insulin antibody (shown in brown), and glucagon antibody (shown in brown) in E. Scale bars: 20 μm. (**F**–**I**), The pancreatic β-cell line MIN6 was transfected with either control (siCont) or siGls2 for 36 hours, and then Gls2 protein level (F), *Gls2* gene expression using RT–qPCR (G), intracellular ATP levels (H) and insulin secretion from the cells to the medium (I) were determined. The blot was cut at 30 kDa before hybridization with β-actin antibody. Data are shown as the mean ± SEM. *P<0.05.

To further elucidate the pathological condition, we then performed a histochemical analysis of the mice to examine insulin and glucagon in pancreatic islets. First, immunostaining of the pancreatic islets showed that Gls2 was positive in insulin- and glucagon-suspected cells in RIP-Cre mice (Supplementary Fig. 4A), and Gls2 was specifically knocked out in insulin-positive pancreatic β-cells from *Gls2* CKO mice (Fig. 2A, B and Supplementary Fig. 4B). Next, the immunohistochemical analysis of the pancreatic islets for HFD-fed *Gls2* CKO mice demonstrated a decrease in the insulin-positive β-cell mass and an increase in the glucagon-positive α-cell mass as well as disorientation of α-cells (Fig. 2C, D and E). The pancreatic islet area did not differ between HFD-fed *Gls2* CKO mice and HFD-RIP-Cre mice. These results indicate the possibility that Gls2 in pancreatic β-cells may affect not only β-cells but also α-cell function. Next, we used the pancreatic β-cell line MIN6 to examine the roles of GLS2 in β cells. First, we confirmed that GLS2 was expressed at protein and mRNA levels in MIN6 (Fig. 2F and G). Since, previous studies have reported that GLS2 promotes ATP production^1^, and intracellular ATP is associated with glucose-stimulated insulin secretion^13^, we examined the effect of GLS2 on ATP level and insulin secretion using Gls2 silenced MIN6 cells. Consistent with previous reports, Gls2 silencing in MIN6 clearly showed lower intracellular ATP levels and lower insulin secretion from cells to the medium compared to cells expressing control RNAi (Fig. 2G, H and I).

### Human type 2 diabetic individuals with low expression of GLS2 showed low insulin gene expression and a low ATP-dependent insulin secretion pathway in β-cells but high glucagon gene expression in α-cells

**Figure 3:**
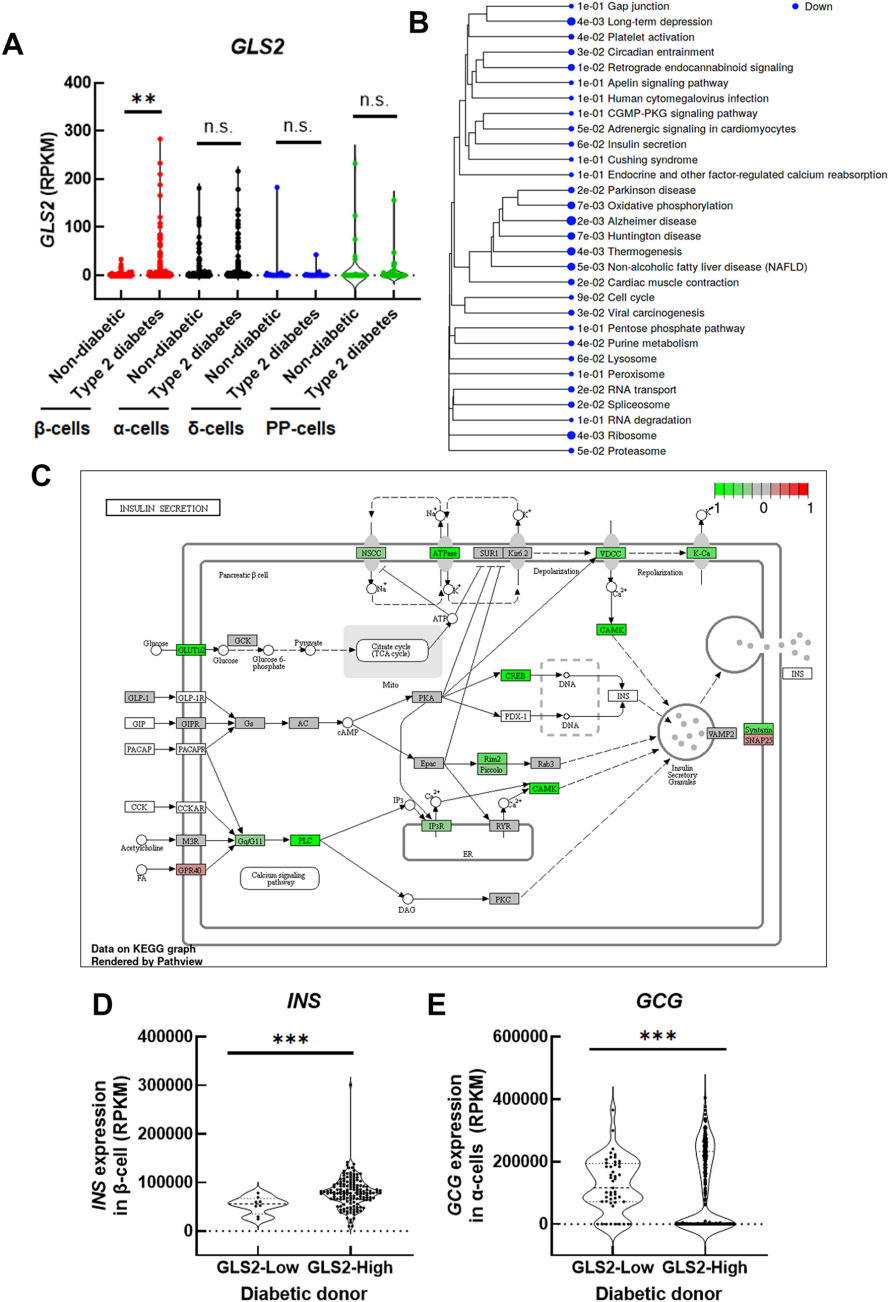
Analysis of *GLS2*, *insulin (INS)* and *glucagon (GCG)* gene expression using a single-cell RNA-sequencing database of human islet cells from type 2 diabetic and nondiabetic donors. (**A**) Violin plots of *GLS2* gene expression in islet cells, including β-cells, α-cells, δ-cells and PP-cells, from type 2 diabetic and nondiabetic donors were assessed using the NCBI database GSE81608^14^. **P<0.01 and ***P<0.001. n.s., not significant. (**B**) Results of the top 30 downregulated pathways in β-cells lacking *GLS2* expression compared to β-cells with *GLS2* expression from type 2 diabetic donors by GAGE analysis using iDEP (integrated Differential Expression and Pathway analysis). (**C**), Expression profiles of the differentially expressed genes in the insulin secretion pathway visualized on the Kyoto Encyclopedia of Genes and Genomes (KEGG) pathway diagram. Red and green indicate genes induced or suppressed in β-cells without *GLS2* expression compared to β-cells with *GLS2* expression, respectively. (**D**–**E**), Comparison of the violin plots of *INS* (**D**) and *GCG* (**E**) gene expression between diabetic donors with the lowest *GLS2* expression and diabetic donors with the highest *GLS2* expression. ***P<0.001.

To investigate whether the regulation of glucose homeostasis in mice was also observed in humans, the human single-cell RNA-sequencing database consisting of 1,600 human islet β-, α-, δ-, and PP-cells from nondiabetic and type 2 diabetes donors was analyzed^14^. The *insulin (INS)* gene was only expressed in pancreatic β-cells, and the *glucagon (GCG)* gene was only expressed in pancreatic α-cells in both nondiabetic and type 2 diabetes donors (Supplementary Fig. 5A and B). On the other hand, the *GLS2* gene was expressed not only in β-cells but also in non-β-cells, including α-cells, consistent with mouse islets. However, the expression of the *GLS2* gene in β-cells was significantly increased in type 2 diabetes donors compared to nondiabetic donors but not in other non-β-cells (Fig. 3A). Next, we compared the differentially expressed gene (DEG) pattern using online application of iDEP (integrated differential expression and pathway analysis) between β-cells lacking *GLS2* gene expression and β-cells with *GLS2* gene expression in diabetic donors. Kyoto Encyclopedia of Genes and Genomes (KEGG) pathway analysis with generally applicable gene set enrichment (GAGE) revealed that the oxidative phosphorylation pathway and insulin secretion pathway, including ATPase and several signaling pathways to insulin secretory granules (https://www.genome.jp/pathway/hsa04911), were significantly reduced in β-cells lacking *GLS2* (Fig. 3B, C and Supplementary Table 1). Further, we compared *INS* and *GCG* gene expression between the donor who showed the lowest *GLS2* expression and the donor with the highest *GLS2* expression in β-cells (Supplementary Fig. 5C). Donors with the lowest *GLS2* expression clearly had lower *INS* and higher *GCG* gene expression than donors with the highest *GLS2* expression (Fig. 3D and E). These results indicate that GLS2 in β-cells is associated with insulin and glucagon organization not only in mice but also in human islet cells.

## Discussion

In this study, we revealed that GLS2 in pancreatic β-cells plays essential roles in glucose homeostasis under a HFD. The fact that Gls2 protein from isolated pancreatic islets increased after HFD feeding along with p53 and 8-OHdG in RIP-Cre mice supported the evidence that GLS2 is a p53 target gene^1,2^ and is activated by p53 possibly through oxidative stress^15^. Pancreatic islet cells, especially β-cells are known to be sensitive to oxidative stress^16^. Furthermore, we found that impairment of Gls2 expression in pancreatic β-cells during hyperglycaemia resulted in the development of significant diabetes mellitus via impairment of insulin increase along with a paradoxical increase in glucagon despite the high plasma glucose. Further, analysis of the human single-cell RNA-sequencing database showed that *GLS2* gene expression was significantly increased in β-cells from diabetic donor with lower *insulin* gene expression but was associated with higher *glucagon* gene expression in α-cells. Our findings present a step forward in explaining one of the critical mechanisms of pancreatic GLS2 in preventing diabetes mellitus.

The present study has some limitations. First, the regulatory mechanism of insulin downregulation by GLS2 was not elucidated in our study. Some reports suggested that glutamate (a GLS2 metabolite) is known to amplify glucose-stimulated insulin secretion in pancreatic β-cells by producing ATP^8^ and by regulating insulin-containing secretory granules^9–12^. Intracellular glutamate increase glucose-stimulated insulin secretion by producing ATP from NADH and NADPH, and fatty acyl-CoA from α-ketoglutarate^17–20^. Glutamate is also involved in exocytosis and proinsulin to insulin conversion by acidifying insulin-containing secretory granules^10–12^. Incretins enhance glucose-stimulated insulin secretion by transporting intracellular glutamate into secretory granules that contain insulin^9^. Moreover, it has been reported that γ-aminobutyric acid (GABA) generated from glutamate increase insulin secretion through activation of GABA receptors in β-cell^21^, and other studies have shown that glutathione peroxidase 1, an antioxidant enzyme important for insulin secretion, uses glutathione generated from glutamate^22,23^. In line with these reports, our results demonstrated that pancreatic β-cell lines transfected with GLS2 silencing showed both low intracellular ATP and low insulin secretion. Additionally, the single-cell RNA-sequencing database demonstrated that the levels of not only ATPase but also several signals to insulin secretory granules decreased in GLS2-downregulated human β-cells. Those data might support the association between GLS2 and ATP or insulin-containing secretory granules in β-cells. Second, it is not clear how GLS2 promoted glucagon secretion. Glucagon is released from pancreatic α-cells and counteracts the glucose-lowering actions of insulin by stimulating gluconeogenesis and hepatic glucose output^24^. However, abnormal glucagon secretion is sometimes observed in diabetes mellitus^24^, and its regulatory mechanism is not fully understood. Glucagon is not only regulated by α-cell-intrinsic glucose sensing but is precisely controlled by the other regulatory mechanisms, including various nutrients, autonomic nerves, the endocrine system, and also the paracrine β-cell effects of insulin^25–27^, GABA^28,29^, and Zn^2+^^24,30^ in the islets. Especially, a paracrine effect between β-cell GLS2/insulin and α-cell glucagon might exist. Future investigation including assessment of the interaction between β-cells and α-cells and their apoptosis/proliferation features are essential to clarifying these pathological mechanisms. Last, there are many uncertainties about GLS2 impairment in diabetes. Genome-wide association studies (GWAS) have reported that variations in loci near the *GLS2* gene are related to glycemic traits, such as fasting glucose, 1-hour glucose, and 2-hour glucose^31^, so clarification of the SNPs in *GLS2* associated with the development of diabetes is desired.

In conclusion, GLS2 impairment in pancreatic β-cells induces insulin impairment and paradoxical glucagon increase, resulting in diabetes mellitus. GLS2, the glutaminolysis key regulator in pancreatic β-cells, maintains glucose homeostasis under the condition of hyperglycaemia.

## Methods

### Mice

Animals were cared for following the guidelines of Chiba University. All of the animal experiments were approved by the Chiba University Review Board for Animal Care. The present study was reported in accordance with the ARRIVE 2.0 Essential 10 guidelines (https://arriveguidelines.org). Pancreatic β-cell-specific *Gls2* conditional knockout mice (CKO) were constructed from rat insulin II promoter (RIP)-Cre transgenic mice [9]. By using a gene targeting strategy containing FLP and loxP, *Gls2* exon 2 through exon 7 in pancreatic β-cells was knocked out. Mice were reared in a pathogen-free environment. From 6 weeks of age, *Gls2* CKO and RIP-Cre mice were raised under a 60 kcal% high-fat diet (#D12492, Research Diets, NJ, USA) up to a maximum of 32 weeks. For mouse genotyping, the primers used for sequencing RIP, FLP, and Neo are listed below. RIP: 5’-ACCTGAAGATGTTCGCGATTATCT-3’ and 5’-ACCGTCAGTACGTGAGATATCTT-3’, FLP1: 5’-TATGTGCCTACTAACGCTTGT-3’ and 5’-AGAGCCACATTCATGAGCTAT-3’, mGls2 dNeo: 5’-CACCTGAGTGAGGTAGACAATCCTA-3’ and 5’-GTAGAAAAGCAGGTCACCAAGTCG-3’.

### Cell culture

A pancreatic β-cell line of MIN6 cells, supplied by ATCC, were cultured in 25 mM glucose (HG)-Dulbecco’s modified Eagle’s medium (DMEM) supplemented with 10% fetal bovine serum (FBS) and 71 μmol/l 2-mercaptoethanol (Sigma).

### Provocation tests and hormonal assays

The provocation tests were performed following a 16-hour fast. The oral glucose loading test (OGTT), insulin tolerance test (ITT), and pyruvate tolerance test (PTT) were performed by orally loading glucose (1 g/kg body weight, Dextrose, Anhydrous, Wako Pure Chemical Industries, LTD, Osaka, Japan), intraperitoneally injecting insulin (0.75 U/kg body weight, Humulin R, Eli Lilly, Indianapolis, In, USA), and intraperitoneally injecting pyruvate (2 g/kg body weight, 20% sodium pyruvate, FUJIFILM Wako Pure Chemical Co., Osaka, Japan), respectively. Blood glucose levels, plasma insulin levels, and plasma glucagon levels were measured by using a glucose monitor (Glutest Mint, Sanwa Kagaku Kenkyusho Co., LTD, Aichi, Japan), an Ultra Sensitive Mouse Insulin ELISA Kit (Morinaga Institute of Biological Science Inc, Kanagawa, Japan), and a Mercodia Glucagon ELISA-10 μL Kit (Mercodia AB, Uppsala, Sweden), respectively.

### Isolation of mouse pancreatic islets

A combination anesthetic was prepared with 0.3 mg/kg of medetomidine, 4.0 mg/kg of midazolam, and 5.0 mg/kg of butorphanol. The anesthetics were administered to mice by intraperitoneal injection, and the islets were isolated from the mouse pancreas using Collagenase P (Cat. No. 11 249 002 001, Roche, Basel, Switzerland) by following the manufacturer’s protocol with slight modification^32^.

### RNA isolation and quantitative RT–qPCR

Total RNA was extracted from each sample, followed by reverse transcription as previously described^33^. The cDNA products were then analyzed by RT–qPCR using the 7500 Fast Real-Time PCR system (Applied Biosystems, Waltham, MA, USA) with the primers listed below. The values of gene-specific mRNA expression were all normalized to the internal housekeeping gene 18S. m*18S*: 5’-CTTAGAGGGACAAGTGGCG-3’ and 5’-ACGCTGAGCCAGTCAGTGTA-3’, m*Gls2*: 5’-AACTATGACAACCTGCGGC-3’　and 5’-GCCGACAATGCAAACCTTC-3’.

### Immunoblotting analysis and antibodies

Immunoblotting analysis was performed as previously described^1^. GLS2, p53 and β-actin proteins were detected by anti-GLS2 antibody (ab113509, Abcam), anti-p53 antibody (FL-393: sc-6243, Santa Cruz) and anti-β-actin antibody (A1978, Sigma), respectively.

### Immunohistochemical and image analysis

Immunohistochemical methods were applied to formalin-fixed paraffin-embedded tissues. The insulin antibody and glucagon antibody were obtained from Santa Cruz Biotechnology (sc-9168 and sc-514592, respectively). The Gls2 and 8-OHdG antibodies were obtained from Gene Tex (GTX31828) and the Japan Institute for the Control of Aging (MOG), respectively. Histochemical image analysis of β-cell/islet and α-cell/islet area were performed using a Keyence Fluorescence Microscope BZ-X700. Histochemical image analysis of Gls2 and 8-OHdG were performed using ImageJ (National Institute of Health).

### Measurement of cellular ATP levels

Intracellular ATP was measured by a luciferin-luciferase bioluminescence assay using the ATP assay reagent (Toyo-ink). In brief, 100 μL of ATP reagent was injected into 96-well samples, and its relative light intensity was recorded in a Lumat LB 9501 luminometer (Berthold) at room temperature.

### Human data acquisition and data analysis

The raw results of single-cell RNA-sequencing data of human pancreas islets were downloaded from the National Center for Biotechnology Information (NCBI) Gene Expression Omnibus (GEO) for GSE81608^14^. Generally applicable gene set enrichment (GAGE)^34^ using integrated differential expression and pathway analysis (iDEP)^35^ was applied for differential pathway enrichment analysis between β-cells with and without *GLS2* expression. GAGE ignores gene sets that contain fewer than 15 genes or more than 2000 genes, and FDR<0.2 was considered. KEGG pathways^36^ were used as input gene sets for GAGE. For each of the significant KEGG pathways, we viewed the fold-changes in related genes on a pathway diagram using the Pathview Bioconductor package^37^.

### Statistics

The results are shown as the mean ± SEM. Two-group comparisons were analyzed by the Mann–Whitney test or Wilcoxon test. All experimental measurements were taken from different individual samples and not repeated measurements of the same sample. Experiments were conducted several times, and representative data are shown.

### Ethics

All animal studies were conducted in accordance with the International Guiding Principles for Biomedical Research Involving Animals and were approved by the Animal Care and Use Committees of Chiba University and the National Institute for Physiological Sciences in Japan.

## Supplementary Information

Supplementary Information 1.

Supplementary Information 2.

Supplementary Information 3.

Supplementary Information 4.

Supplementary Information 5.

Supplementary Information 6.

## Data Availability

The data produced during the current study and materials are available from the corresponding author upon reasonable request.
