# Supplementary figures and images for: Pancreatic β-cell glutaminase 2 maintains glucose homeostasis under the condition of hyperglycaemia
Source: Sci Rep. 2023 May 5;13:7291. doi: 10.1038/s41598-023-34336-z (PMC10162969; doi:10.1038/s41598-023-34336-z)

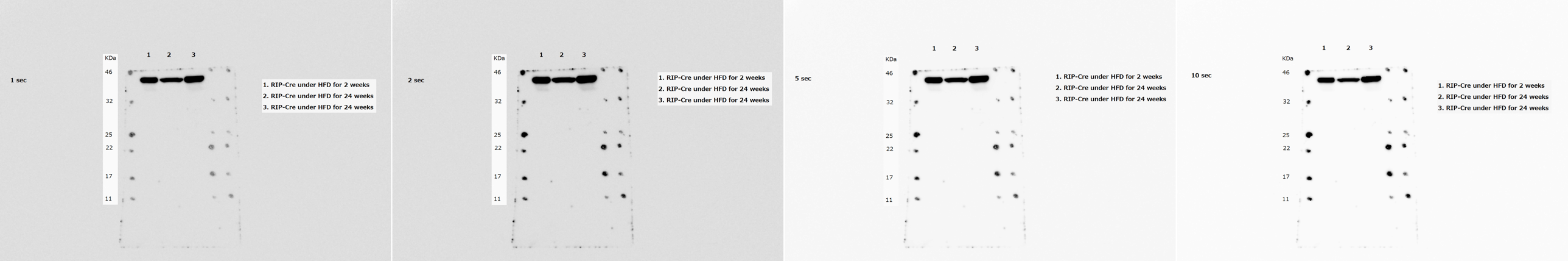

Supplement: Supplementary file 3 — Supplementary Information 3. [file 41598_2023_34336_MOESM3_ESM.jpg]

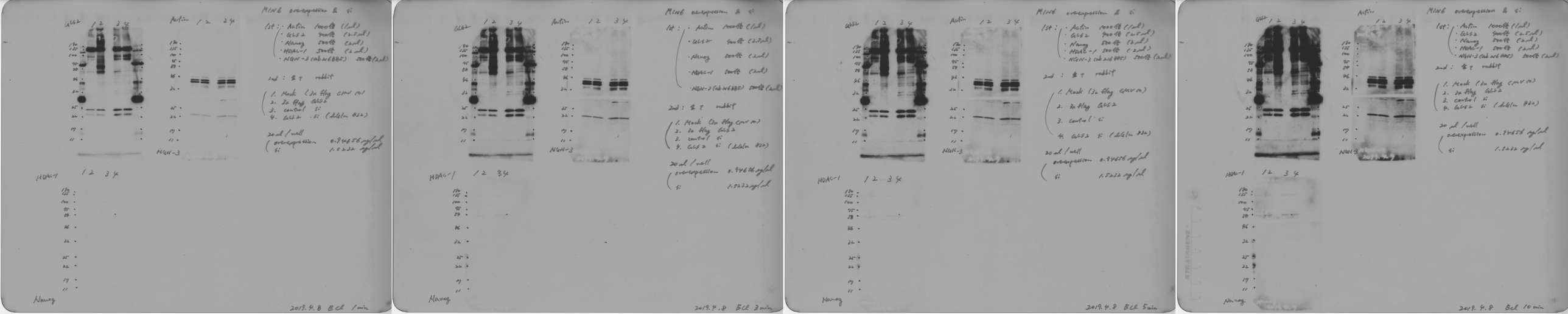

Supplement: Supplementary file 5 — Supplementary Information 5. [file 41598_2023_34336_MOESM5_ESM.jpg]
