# Supplementary material for: Pancreatic β-cell glutaminase 2 maintains glucose homeostasis under the condition of hyperglycaemia
Source: Sci Rep. 2023 May 5;13:7291. doi: 10.1038/s41598-023-34336-z (PMC10162969; doi:10.1038/s41598-023-34336-z)
Supplement: Supplementary file 6 — Supplementary Information 6. [file 41598_2023_34336_MOESM6_ESM.docx]

Supplementary Information for

**Pancreatic β-cell glutaminase 2 maintains glucose homeostasis**

**under the condition of hyperglycaemia.**

Hanna Deguchi-Horiuchi^1)2)^, Sawako Suzuki^1)2)＊^, Eun Young Lee ^3)^, Takashi Miki^3)^, Noriko Yamanaka^4)^, Ichiro Manabe^4)^, Tomoaki Tanaka^5)^ and Koutaro Yokote^1)2)^

^1)^ Department of Endocrinology, Hematology and Gerontology, Graduate School of Medicine, Chiba University, Chiba, Japan

^2)^ Department of Diabetes, Metabolism and Endocrinology, Chiba University hospital, Chiba, Japan

^3)^ Department of Medical Physiology, Graduate School of Medicine, Chiba University, Chiba, Japan

^4)^ Department of Disease Biology and Molecular Medicine, Graduate School of Medicine, Chiba University, Chiba, Japan

^5)^ Department of Molecular Diagnosis, Graduate School of Medicine, Chiba University, Chiba, Japan

＊Correspondence to: Sawako Suzuki, Department of Diabetes, Metabolism and Endocrinology, Chiba University Hospital, 1-8-1 Inohana Chuo-ku Chiba 260-8670, Japan E-mail: [sawakosuzuki@chiba-u.jp](mailto:sawakosuzuki@chiba-u.jp)

Short running title: Glutaminase 2 in pancreatic β-cell.

Key words: Glutaminase 2, pancreatic β-cells, insulin, glucagon, glucose homeostasis.

This word file includes: Supplementary Table 1, Supplemental Figure 1-5.

**Supplementary Table 1. Top 20 KEGG pathways decreased in pancreatic β-cells without *Gls2* compared to β-cells with *Gls2*.**

|  | Direction | Pathways | Number of genes | P-value | Genes |
| --- | --- | --- | --- | --- | --- |
| 1 | Down | Alzheimer disease | 132 | 0.0015 | ADAM10 CDK5 RTN3 ATP5PD UQCR11 COX6B2 NDUFA11 COX4I1 COX5B COX6A1 COX6A2 COX6B1 COX6C COX7A1 COX7A2 COX7C COX8A CYC1 ERN1 ATF6 PLCB1 NCSTN BACE1 BACE2 GAPDH UQCRQ GNAQ GRIN2A GSK3B UQCR10 HSD17B10 APAF1 APBB1 IDE COX8C APOE FAS ITPR1 ITPR2 ITPR3 NDUFS7 LPL LRP1 UQCRHL MT-ATP8 MT-CO1 MT-CO3 MT-CYB NDUFA1 NDUFA2 NDUFA4 NDUFA5 NDUFA7 NDUFA8 NDUFA9 NDUFA10 NDUFAB1 NDUFB1 NDUFB2 NDUFB3 NDUFB4 NDUFB5 NDUFB6 NDUFB9 NDUFB10 NDUFC1 NDUFC2 NDUFS1 NDUFS2 NDUFV1 NDUFS4 NDUFS5 NDUFS6 NDUFS8 NDUFV3 NOS1 ATP2A1 ATP2A2 ATP2A3 ATP5F1A ATP5F1B ATP5F1C NDUFA13 APH1A ATP5F1D ATP5F1E ATP5PB ATP5MC1 ATP5MC2 PLCB2 PLCB4 ATP5PO NDUFB11 PPP3CA PPP3CB PPP3CC PPP3R1 MAPK1 MAPK3 PSEN1 PSEN2 RTN4 BAD RYR3 BID SDHA SDHB SDHC SDHD TNFRSF1A UQCRB UQCRC1 UQCRC2 UQCRFS1 UQCRH CACNA1C CACNA1D CACNA1S CALM1 CALM2 CALM3 CAPN1 CAPN2 APH1B CASP7 CASP8 CASP9 FADD CDK5R1 NAE1 COX7A2L COX5A |
| 2 | Down | Long-term depression | 36 | 0.0036 | CRH PLCB1 GNA11 GNAI1 GNAI2 GNAI3 GNAO1 GNAQ GNAS GNAZ GRIA2 GRIA3 GRID2 GRM1 GUCY1A1 GUCY1B1 ARAF ITPR1 ITPR2 ITPR3 LYN NOS1 PLA2G4A PLCB2 PLCB4 PPP2CA PPP2CB PPP2R1A PRKCA PRKG2 MAPK1 MAPK3 MAP2K1 RAF1 CACNA1A PLA2G4C |
| 3 | Down | Thermogenesis | 177 | 0.0036 | COX17 ATP5PD CREB3 ATP5MG ADCY1 FRS2 PPARGC1A UQCR11 ADCY5 MGLL COX20 SLC25A29 COX6B2 CPT1C NDUFA11 CNR1 COX4I1 COX5B COX6A1 COX6A2 COX6B1 COX6C COX7A1 COX7A2 COX7C COX8A COX10 COX11 COX15 CPT1A CPT1B CPT2 NDUFAF6 CREB1 ATF2 MAPK14 CREB3L4 KLB CYC1 ACSL4 FGFR1 KDM1A ACSL6 MTOR GCG UQCRQ RPS6KA6 GNAS COX18 COA3 NDUFAF4 UQCR10 COX8C BMP8A NDUFS7 COA6 LIPE MAP3K5 UQCRHL MT-ATP8 MT-CO1 MT-CO3 MT-CYB MT-ND1 MT-ND2 MT-ND3 MT-ND4 MT-ND4L MT-ND5 MT-ND6 NDUFA1 NDUFA2 NDUFA4 NDUFA5 NDUFA7 NDUFA8 NDUFA9 NDUFA10 NDUFAB1 NDUFB1 NDUFB2 NDUFB3 NDUFB4 NDUFB5 NDUFB6 NDUFB9 NDUFB10 NDUFC1 NDUFC2 NDUFS1 NDUFS2 NDUFV1 NDUFS4 NDUFS5 NDUFS6 NDUFS8 NDUFV3 NPPA COA5 ATP5F1A ATP5F1B ATP5F1C NDUFA13 COX16 COA4 ATP5F1D ATP5F1E ACTL6B PRKAG2 ATP5PB SIRT6 ATP5MC1 ATP5MC2 KDM3B ATP5ME PLIN1 PRKAG3 ATP5PO NDUFB11 PPARG NDUFAF7 PRKAB1 PRKAB2 PRKACA PRKAG1 COA1 KDM3A PRKG2 MAPK11 MAP2K3 ARID1B ACTB RHEB RPS6 RPS6KA1 RPS6KA2 RPS6KA3 RPS6KB1 MAPK12 SDHA SDHB SDHC SDHD PRDM16 MLST8 CREB3L2 BMP8B SMARCA2 SMARCB1 SMARCC1 SMARCC2 SMARCD1 SMARCD2 SMARCD3 SMARCE1 SOS1 SOS2 ACTG1 TSC1 TSC2 UQCRB UQCRC1 UQCRC2 UQCRFS1 UQCRH SLC25A20 NDUFAF5 DPF3 DPF1 ARID1A COX14 ACTL6A COX19 COX7A2L COX5A ATP5MF CREB5 |
| 4 | Down | Ribosome | 107 | 0.0036 | RPL36A-HNRNPH2 RPS10-NUDT3 MRPL28 MRPL3 RPL35 MRPL10 RPS4Y2 RPL22L1 FAU MRPL21 RPL13A RPL36 MRPL13 MRPL18 MRPL15 MRPL22 RPSA RPL10A MRPS16 MRPS18C RPS27L MRPL2 MRPL4 MRPS2 RPL26L1 RSL24D1 MRPL30 MRPL27 MRPL35 MRPS17 MRPL16 MRPS18A MRPS10 RPL3 RPL3L RPL5 RPL6 RPL7 RPL8 RPL9 RPL11 RPL12 RPL13 RPL15 RPL18 RPL18A RPL19 RPL21 RPL22 MRPL23 RPL24 RPL26 RPL27 RPL27A RPL28 RPL29 RPL31 RPL34 RPL35A RPL36AL RPL37 RPL37A RPL38 RPL39 RPL36A RPLP0 RPLP1 MRPS12 RPS3 RPS3A RPS4X RPS4Y1 RPS5 RPS6 RPS7 RPS8 RPS10 RPS11 RPS12 RPS13 RPS14 RPS15 RPS15A RPS16 RPS19 RPS20 RPS24 RPS25 RPS27 RPS27A RPS28 RPS29 MRPL17 MRPS14 MRPL14 MRPS15 MRPS11 MRPS9 MRPS6 MRPS5 MRPL36 MRPL34 MRPL32 MRPL9 MRPL24 RPL14 MRPL33 |
| 5 | Down | Non-alcoholic fatty liver disease (NAFLD) | 119 | 0.0054 | BCL2L11 NDUFC2-KCTD14 NR1H3 CEBPA UQCR11 COX6B2 NDUFA11 COX4I1 COX5B COX6A1 COX6A2 COX6B1 COX6C COX7A1 COX7A2 COX7C COX8A CYC1 CYP2E1 DDIT3 EIF2S1 AKT1 AKT2 ERN1 MLXIP UQCRQ GSK3A GSK3B UQCR10 COX8C FAS IKBKB IL1A CXCL8 IRS1 JUN NDUFS7 LEPR MAP3K5 UQCRHL MT-CO1 MT-CO3 MT-CYB ATF4 NDUFA1 NDUFA2 NDUFA4 NDUFA5 NDUFA7 NDUFA8 NDUFA9 NDUFA10 NDUFAB1 NDUFB1 NDUFB2 NDUFB3 NDUFB4 NDUFB5 NDUFB6 NDUFB9 NDUFB10 NDUFC1 NDUFC2 NDUFS1 NDUFS2 NDUFV1 NDUFS4 NDUFS5 NDUFS6 NDUFS8 NDUFV3 NFKB1 NDUFA13 MLXIPL ADIPOR1 PRKAG2 PIK3CA PIK3CB PIK3CD PIK3R1 PIK3R2 PRKAG3 NDUFB11 PPARA PRKAB1 PRKAB2 PRKAG1 MAPK8 MAPK9 MAPK10 BAX RAC1 RELA RXRA BID SDHA SDHB SDHC SDHD SREBF1 MLX ENSG00000105329 TNFRSF1A TRAF2 UQCRB UQCRC1 UQCRC2 UQCRFS1 UQCRH XBP1 ADIPOR2 ITCH CASP7 CASP8 PIK3R3 SOCS3 COX7A2L COX5A EIF2AK3 |
| 6 | Down | Oxidative phosphorylation | 112 | 0.0067 | COX17 TCIRG1 ATP5PD ATP5MG UQCR11 COX6B2 NDUFA11 COX4I1 COX5B COX6A1 COX6A2 COX6B1 COX6C COX7A1 COX7A2 COX7C COX8A COX10 COX11 COX15 CYC1 ATP6V0E2 ATP6V0A2 ATP6V0D2 ATP6V1C2 PPA2 UQCRQ UQCR10 COX8C NDUFS7 UQCRHL MT-ATP8 MT-CO1 MT-CO3 MT-CYB MT-ND1 MT-ND2 MT-ND3 MT-ND4 MT-ND4L MT-ND5 MT-ND6 NDUFA1 NDUFA2 NDUFA4 NDUFA5 NDUFA7 NDUFA8 NDUFA9 NDUFA10 NDUFAB1 NDUFB1 NDUFB2 NDUFB3 NDUFB4 NDUFB5 NDUFB6 NDUFB9 NDUFB10 NDUFC1 NDUFC2 NDUFS1 NDUFS2 NDUFV1 NDUFS4 NDUFS5 NDUFS6 NDUFS8 NDUFV3 ATP5F1A ATP5F1B ATP6V0A4 ATP5F1C NDUFA13 ATP5F1D ATP6V1D ATP5F1E ATP5PB ATP5MC1 ATP6V1H ATP5MC2 ATP5ME ATP6V1A ATP6V1B1 ATP6V1B2 ATP6V0C ATP6V1C1 ATP6V1E1 ATP6V0B ATP6V1G2 ATP6V0A1 ATP6AP1 ATP5PO NDUFB11 PPA1 SDHA SDHB SDHC SDHD LHPP UQCRB UQCRC1 UQCRC2 UQCRFS1 UQCRH ATP6V0E1 ATP6V1E2 ATP6V0D1 COX7A2L ATP6V1F COX5A ATP6V1G1 |
| 7 | Down | Huntington disease | 149 | 0.0072 | PPIF DNAL4 ATP5PD CREB3 DCTN2 PPARGC1A UQCR11 AP2S1 CLTA CLTB COX6B2 NDUFA11 DNAH14 COX4I1 COX5B COX6A1 COX6A2 COX6B1 COX6C COX7A1 COX7A2 COX7C COX8A CREB1 CREBBP DNAH2 CREB3L4 CYC1 AP2A2 AP2B1 DCTN1 DLG4 DNAH5 DNAH6 DNAH8 DNAH9 DNAH12 EP300 RCOR1 PLCB1 POLR2J2 SIN3A DNAH1 DNAI1 UQCRQ BBC3 GNAQ SLC25A4 SLC25A5 UQCR10 HTT HDAC1 HDAC2 HIP1 APAF1 COX8C ITPR1 NDUFS7 TBPL2 UQCRHL MT-ATP8 MT-CO1 MT-CO3 MT-CYB NDUFA1 NDUFA2 NDUFA4 NDUFA5 NDUFA7 NDUFA8 NDUFA9 NDUFA10 NDUFAB1 NDUFB1 NDUFB2 NDUFB3 NDUFB4 NDUFB5 NDUFB6 NDUFB9 NDUFB10 NDUFC1 NDUFC2 NDUFS1 NDUFS2 NDUFV1 NDUFS4 NDUFS5 NDUFS6 NDUFS8 NDUFV3 NRF1 ATP5F1A ATP5F1B ATP5F1C NDUFA13 ATP5F1D ATP5F1E ATP5PB ATP5MC1 ATP5MC2 PLCB2 PLCB4 ATP5PO POLR2B POLR2C POLR2D POLR2E POLR2F POLR2H POLR2I POLR2J POLR2K POLR2L NDUFB11 PPARG POLR2J3 IFT57 DNAH7 BAX REST BDNF SDHA SDHB SDHC SDHD DNAI2 CREB3L2 SP1 TAF4B TBP TFAM TGM2 TP53 UQCRB UQCRC1 UQCRC2 UQCRFS1 UQCRH VDAC2 VDAC3 DNALI1 CLTCL1 DNAL1 CASP8 CASP9 COX7A2L COX5A TBPL1 |
| 8 | Down | Retrograde endocannabinoid signaling | 102 | 0.013 | GNB5 ADCY1 ADCY5 MGLL NDUFA11 CNR1 MAPK14 FAAH DAGLB RIMS1 PLCB1 GABRA1 GABRA2 GABRA3 GABRB1 GABRB2 GABRB3 GABRG2 GABRP GNAI1 GNAI2 GNAI3 GNAO1 GNAQ GNB1 GNB2 GNB3 GNG4 GNG5 GNG7 GNG10 GNG11 GNGT1 GRIA2 GRIA3 GRIA4 GRM1 ITPR1 ITPR2 ITPR3 NDUFS7 KCNJ3 KCNJ5 MT-ND1 MT-ND2 MT-ND3 MT-ND4 MT-ND4L MT-ND5 MT-ND6 NDUFA1 NDUFA2 NDUFA4 NDUFA5 NDUFA7 NDUFA8 NDUFA9 NDUFA10 NDUFAB1 NDUFB1 NDUFB2 NDUFB3 NDUFB4 NDUFB5 NDUFB6 NDUFB9 NDUFB10 NDUFC1 NDUFC2 NDUFS1 NDUFS2 NDUFV1 NDUFS4 NDUFS5 NDUFS6 NDUFS8 NDUFV3 NDUFA13 PLCB2 PLCB4 GNG2 NDUFB11 PRKACA PRKCA MAPK1 MAPK3 GNG12 MAPK8 MAPK11 MAPK9 MAPK10 SLC17A6 ABHD6 PTGS2 GNB4 MAPK12 DAGLA CACNA1A CACNA1B CACNA1C CACNA1D CACNA1S |
| 9 | Down | Cardiac muscle contraction | 57 | 0.02 | CACNG2 SLC9A6 UQCR11 COX6B2 COX4I1 COX5B COX6A1 COX6A2 COX6B1 COX6C COX7A1 COX7A2 COX7C COX8A CYC1 UQCRQ CACNG5 CACNG4 UQCR10 COX8C UQCRHL MT-CO1 MT-CO3 MT-CYB MYH7 ATP1A1 ATP1A3 ATP1B1 ATP1B2 ATP1B3 FXYD2 ATP2A2 CACNA2D3 CACNG8 RYR2 SLC8A1 SLC9A1 TNNC1 ENSG00000129991 TPM1 TPM2 TPM3 UQCRB UQCRC1 UQCRC2 UQCRFS1 UQCRH CACNA1C CACNA1D CACNA1S CACNA2D1 CACNB1 CACNB2 CACNB3 CACNB4 COX7A2L CACNA2D2 |
| 10 | Down | Parkinson disease | 110 | 0.022 | PPIF ATP5PD UQCR11 ADCY5 PARK7 UBE2J2 LRRK2 COX6B2 NDUFA11 COX4I1 COX5B COX6A1 COX6A2 COX6B1 COX6C COX7A1 COX7A2 ADORA2A COX7C COX8A CYC1 UQCRQ HTRA2 GNAI1 GNAI2 GNAI3 GNAL SLC25A4 SLC25A5 UQCR10 APAF1 COX8C NDUFS7 UQCRHL MT-ATP8 MT-CO1 MT-CO3 MT-CYB MT-ND1 MT-ND2 MT-ND3 MT-ND4 MT-ND4L MT-ND5 MT-ND6 NDUFA1 NDUFA2 NDUFA4 NDUFA5 NDUFA7 NDUFA8 NDUFA9 NDUFA10 NDUFAB1 NDUFB1 NDUFB2 NDUFB3 NDUFB4 NDUFB5 NDUFB6 NDUFB9 NDUFB10 NDUFC1 NDUFC2 NDUFS1 NDUFS2 NDUFV1 NDUFS4 NDUFS5 NDUFS6 NDUFS8 NDUFV3 ATP5F1A ATP5F1B PRKN ATP5F1C NDUFA13 ATP5F1D ATP5F1E UBE2J1 ATP5PB ATP5MC1 ATP5MC2 ATP5PO NDUFB11 PRKACA SDHA SDHB SDHC SDHD SLC18A1 SLC18A2 TH UBB UBA1 UBE2G1 UBE2G2 UBE2L3 UCHL1 UQCRB UQCRC1 UQCRC2 UQCRFS1 UQCRH VDAC2 VDAC3 CASP9 COX7A2L UBE2L6 COX5A |
| 11 | Down | Spliceosome | 110 | 0.022 | PQBP1 SF3B4 BCAS2 SF3A1 PPIE PPIH CHERP SLU7 PRPF8 USP39 SRSF10 TCERG1 SRSF8 SF3A3 SF3B2 SNRNP27 LSM6 DDX42 U2AF2 HNRNPA1L2 CCDC12 ZMAT2 DDX5 DHX8 DHX15 U2AF1L4 PUF60 NCBP2 SNW1 ACIN1 SNRNP200 U2SURP SF3B1 LSM5 PRPF6 PRPF40B ENSG00000130520 SYF2 PRPF31 LSM3 RBMX TRA2A HNRNPA1 HNRNPC HNRNPU HSPA2 HSPA6 HSPA8 MAGOH HNRNPM NCBP1 SNU13 RBMXL1 CRNKL1 CWC15 SF3B6 PPIL1 LSM7 LSM8 WBP11 PLRG1 MAGOHB PRPF38B PRPF40A CTNNBL1 XAB2 THOC2 ISY1 RBM25 RP9 SRSF1 SRSF2 SRSF4 SRSF5 SRSF6 SRSF7 TRA2B SNRNP70 SNRPA SNRPA1 SNRPB SNRPB2 SNRPC SNRPD1 SNRPD2 SNRPD3 SNRPE SNRPF SNRPG U2AF1 SF3A2 SF3B5 THOC3 PHF5A PRPF38A RBM17 PRPF18 SRSF9 BUD31 SART1 PRPF4 PRPF3 EFTUD2 SNRNP40 DDX23 AQR EIF4A3 DHX38 CDC5L RBM8A |
| 12 | Down | RNA transport | 135 | 0.024 | SNUPN POP7 SRRM1 SAP18 EIF1B PRMT5 TACC3 NXF1 RPP30 RPP38 PAIP1 NUP50 POP4 RPP40 POP1 NUPL2 RPP14 STRAP DDX20 XPOT CLNS1A NUP35 RPP25L PABPC5 EEF1A1 EEF1A2 EIF2S1 EIF2B1 EIF2S3 EIF4A1 EIF4A2 EIF4B EIF4E EIF4EBP1 EIF4EBP2 EIF4G1 EIF4G2 EIF5 CASC3 NCBP2 ACIN1 NUP205 CYFIP1 NUP210 NUP160 FMR1 NUP188 EIF4E1B GEMIN5 PABPC1 CYFIP2 NXT1 NUP43 EIF3E KPNB1 SUMO4 MAGOH NCBP1 NUP88 NUP98 PABPC3 NMD3 TRNT1 POP5 PHAX NUP54 PNN RPP25 GEMIN8 MAGOHB ELAC1 NDC1 NXT2 NUP107 THOC2 XPO5 RAN RANBP2 SENP2 UPF1 ELAC2 SEC13 UPF3B UPF3A SUMO3 SUMO2 TPR EIF3CL SUMO1 XPO1 NUP37 THOC6 GEMIN7 GEMIN6 NUP85 THOC7 NUP214 PABPC1L AAAS FXR1 SEH1L PYM1 THOC3 RAE1 GEMIN2 THOC5 EIF4EBP3 EIF3A EIF3B EIF3D EIF3F EIF3G EIF3H EIF3I EIF3J EIF4G3 PABPC4 EIF2B4 EIF2B3 EIF2B2 EIF2B5 EIF2S2 EIF1AY NUP210L POM121L2 EIF4E2 FXR2 NUP155 EIF5B TGS1 NUP93 EIF4A3 NUP58 RBM8A NUP153 |
| 13 | Down | Circadian entrainment | 58 | 0.029 | ADCY1 ADCY5 CREB1 PLCB1 GNAI1 GNAI2 GNAI3 GNAO1 GNAQ GNAS GNB1 GNB2 GNB3 GNG4 GNG5 GNG7 GNG10 GNG11 GNGT1 GRIA2 GRIA3 GRIA4 GRIN2A GUCY1A1 GUCY1B1 ITPR1 ITPR3 KCNJ3 KCNJ5 NOS1 RASD1 ENSG00000179094 PLCB2 PLCB4 GNG2 PRKACA PRKCA PRKG2 MAPK1 MAPK3 GNG12 GNB4 RYR2 RYR3 CACNA1C CACNA1D CALM1 CALM2 CALM3 CAMK2B CAMK2D CAMK2G PER3 PER2 CACNA1I CACNA1H CACNA1G RPS6KA5 |
| 14 | Down | Viral carcinogenesis | 142 | 0.029 | HDAC5 CDK2 CDK4 CDK6 CDKN1A CDKN1B CDKN2A IRF9 CREB3 CHD4 CHEK1 HIST4H4 CCR8 HIST3H2BB CREB1 ATF2 CREBBP CREB3L4 DDB1 DLG1 EGR2 EGR3 EP300 SNW1 UBR4 ATP6V0D2 KAT2A H2BFM GSN GTF2A1 GTF2A2 GTF2B GTF2E1 GTF2E2 GTF2H1 GTF2H3 HIST1H2BD HDAC1 HDAC2 HLA-C HPN RBPJ IRF3 IRF7 JAK1 JAK3 JUN RHOA TBPL2 LYN MDM2 HIST2H2BF ATF4 NFKB1 NFKB2 NFKBIA HDAC7 PIK3CA PIK3CB PIK3CD PIK3R1 PIK3R2 PKM POLB HIST2H4B PRKACA VAC14 HDAC8 MAPK1 MAPK3 EIF2AK2 PSMC1 BAD BAK1 BAX PXN RAC1 RANBP1 RASA2 RB1 RBL2 CCND1 REL RELA CREB3L2 SP100 STAT3 STAT5A TBP TP53 C3 TRAF1 TRAF2 TRAF3 TRAF5 UBE3A VDAC3 YWHAG YWHAH YWHAZ USP7 HDAC11 HIST1H4I HIST1H2BG HIST1H2BL HIST1H2BN HIST1H2BM HIST1H2BI HIST1H2BC HIST2H2BE HIST1H4D HIST1H4K HIST1H4J HIST1H4C HIST1H4H HIST1H4E HIST2H4A MAD1L1 HDAC10 CASP8 PIK3R3 IKBKG HIST1H2BK SCIN ACTN1 TRADD KAT2B CCNA2 CCNA1 CCND2 CCND3 HIST1H2BJ CCNE1 ATP6V0D1 CCNE2 MAPKAPK2 TBPL1 CREB5 HDAC9 HDAC4 CDK1 CDC20 |
| 15 | Down | Proteasome | 32 | 0.046 | PSMD14 ADRM1 PSME4 POMP PSMA2 PSMA3 PSMA4 PSMA5 PSMA6 PSMA7 PSMB1 PSMB2 PSMB4 PSMB5 PSMB10 PSMC1 PSMC2 PSMC3 PSMC4 PSMC5 PSMC6 PSMD1 PSMD3 PSMD4 PSMD7 PSMD8 PSMD11 PSMD13 PSME1 PSME2 SEM1 PSMF1 |
| 16 | Down | Purine metabolism | 91 | 0.04 | NT5C1B-RDH14 NME6 PAICS ADCY1 PDE10A ADCY5 NUDT5 NT5C3B AK7 ADSSL1 CANT1 NUDT16 ADK ADSL AK8 ADSS AK1 AK2 AK4 AK9 PRPS1L1 FHIT ENPP4 NT5C2 AK5 AMPD1 AMPD2 PDE7B GMPR GUCY1A1 GUCY1B1 GUCY2C GUK1 NME7 GUCY2D NT5C NUDT2 HPRT1 APRT ITPA HDDC3 ATIC NME1 NME2 NME3 NME4 PNP NPR2 NT5E RRM2B NT5C3A GMPR2 PDE1A PDE2A PDE3A PDE3B ENSG00000065989 PDE4B PDE4C PDE4D PDE6C PDE6D PDE8A PDE9A PDE1B PDE6B ENPP1 PFAS PGM1 PKM NUDT9 PPAT PGM2 PRPS1 PRPS2 NT5M ADPRM PRUNE1 RRM1 NME1-NME2 XDH NTPCR PDE5A GMPS PAPSS2 PAPSS1 ENTPD2 ENTPD6 ENTPD3 ENTPD5 ENTPD4 |
| 17 | Down | Platelet activation | 90 | 0.04 | PLA2G4B RASGRP1 MYL12B MYL12A ADCY1 ADCY5 COL1A1 COL1A2 COL3A1 MAPK14 PIK3R6 AKT1 AKT2 F2R FCER1G FCGR2A FGG PLCB1 ARHGEF12 FYN GNAI1 GNAI2 GNAI3 GNAQ GNAS GP1BA GP1BB ARHGAP35 GUCY1A1 GUCY1B1 MYLK4 ITGA2 ITGA2B ITGB1 ITPR1 ITPR2 ITPR3 RHOA LCP2 LYN MYLK PPP1R12A NOS3 P2RX1 P2RY1 PIK3CA PIK3CB PIK3CD PIK3CG PIK3R1 PIK3R2 PLA2G4A PLCB2 PLCB4 PLCG2 APBB1IP PPP1CB PPP1CC PRKACA PRKCI PRKCZ PRKG2 MAPK1 MAPK3 MAPK11 PTGIR PTGS1 RAP1A RAP1B ACTB ROCK1 MAPK12 P2RY12 STIM1 TBXA2R TBXAS1 BTK TLN1 ACTG1 VASP TLN2 FERMT3 ORAI1 PIK3R3 PLA2G4C JMJD7-PLA2G4B SNAP23 F2RL3 ARHGEF1 MYLK3 |
| 18 | Down | Adrenergic signaling in cardiomyocytes | 98 | 0.051 | CACNG2 RAPGEF3 CREB3 ADCY1 RAPGEF4 ADCY5 CREB1 ATF2 CREM MAPK14 PIK3R6 CREB3L4 ADRB1 AGT AGTR1 AKT1 AKT2 PLCB1 CACNG5 CACNG4 GNAI1 GNAI2 GNAI3 GNAQ GNAS KCNQ1 MYH7 ATF4 ATP1A1 ATP1A3 ATP1B1 ATP1B2 ATP1B3 FXYD2 ATP2A2 ATP2B1 ATP2B2 ATP2B3 ATP2B4 PIK3CG PLCB2 PLCB4 PLN PPP1CB PPP1CC PPP2R3C PPP1R1A PPP2CA PPP2CB PPP2R1A PPP2R2A PPP2R2B PPP2R2C PPP2R3A PPP2R5A PPP2R5B PPP2R5C PPP2R5D PPP2R5E PRKACA PRKCA CACNA2D3 PPP2R2D MAPK1 MAPK3 MAPK11 CACNG8 BCL2 RYR2 MAPK12 SCN1B SCN4B SCN5A SCN7A CREB3L2 SLC8A1 SLC9A1 TNNC1 ENSG00000129991 TPM1 TPM2 TPM3 CACNA1C CACNA1D CACNA1S CACNA2D1 CACNB1 CACNB2 CACNB3 CACNB4 CALM1 CALM2 CALM3 CAMK2B CAMK2D CAMK2G RPS6KA5 CACNA2D2 |
| 19 | Down | Insulin secretion | 48 | 0.058 | CREB3 ADCY1 RAPGEF4 ADCY5 CHRM3 CREB1 ATF2 GPR119 CREB3L4 PLCB1 GCG GCK PCLO GNA11 GNAQ GNAS FFAR1 ITPR3 KCNJ11 KCNMA1 KCNN3 ATF4 ATP1A1 ATP1A3 ATP1B1 ATP1B2 ATP1B3 FXYD2 PLCB2 PLCB4 TRPM4 PRKACA PRKCA RAB3A RYR2 CREB3L2 SLC2A1 SNAP25 STX1A ABCC8 VAMP2 CACNA1C CACNA1D CACNA1S CAMK2B CAMK2D CAMK2G CREB5 |
| 20 | Down | Lysosome | 97 | 0.058 | AP3S2 TCIRG1 NPC2 AP4B1 CTSC AP3M2 AP4S1 AP1S1 AP3S1 CLN3 CLN5 CLTA CLTB AP1S3 HGSNAT CTNS CTSD CTSH CTSK CTSW CTSZ AP1B1 AP1G1 AGA DNASE2 GGA2 GGA3 AP4E1 ABCB9 ATP6V0A2 PLA2G15 ATP6V0D2 GAA MFSD8 GALC GALNS GGA1 SLC17A5 AP3M1 LAMP3 GLA GLB1 GNS SUMF1 HEXA HEXB HYAL1 IDS LAMP2 LIPA M6PR ARSA ARSB MAN2B1 MANBA ASAH1 NAGA NAGLU NPC1 SLC11A2 ATP6V0A4 NAGPA ATP6V1H ATP6V0C ACP2 ATP6V0B ATP6V0A1 ATP6AP1 ACP5 CTSA LAPTM4B PPT1 LGMN MCOLN1 SORT1 SGSH SLC11A1 SMPD1 LAPTM5 GNPTAB AP3B2 CLTCL1 GNPTG AP3B1 CD164 AP1S2 AP1G2 AP1M1 AP3D1 ATP6V0D1 AP4M1 NAPSA SCARB2 LITAF ENTPD4 CD63 CD68 |


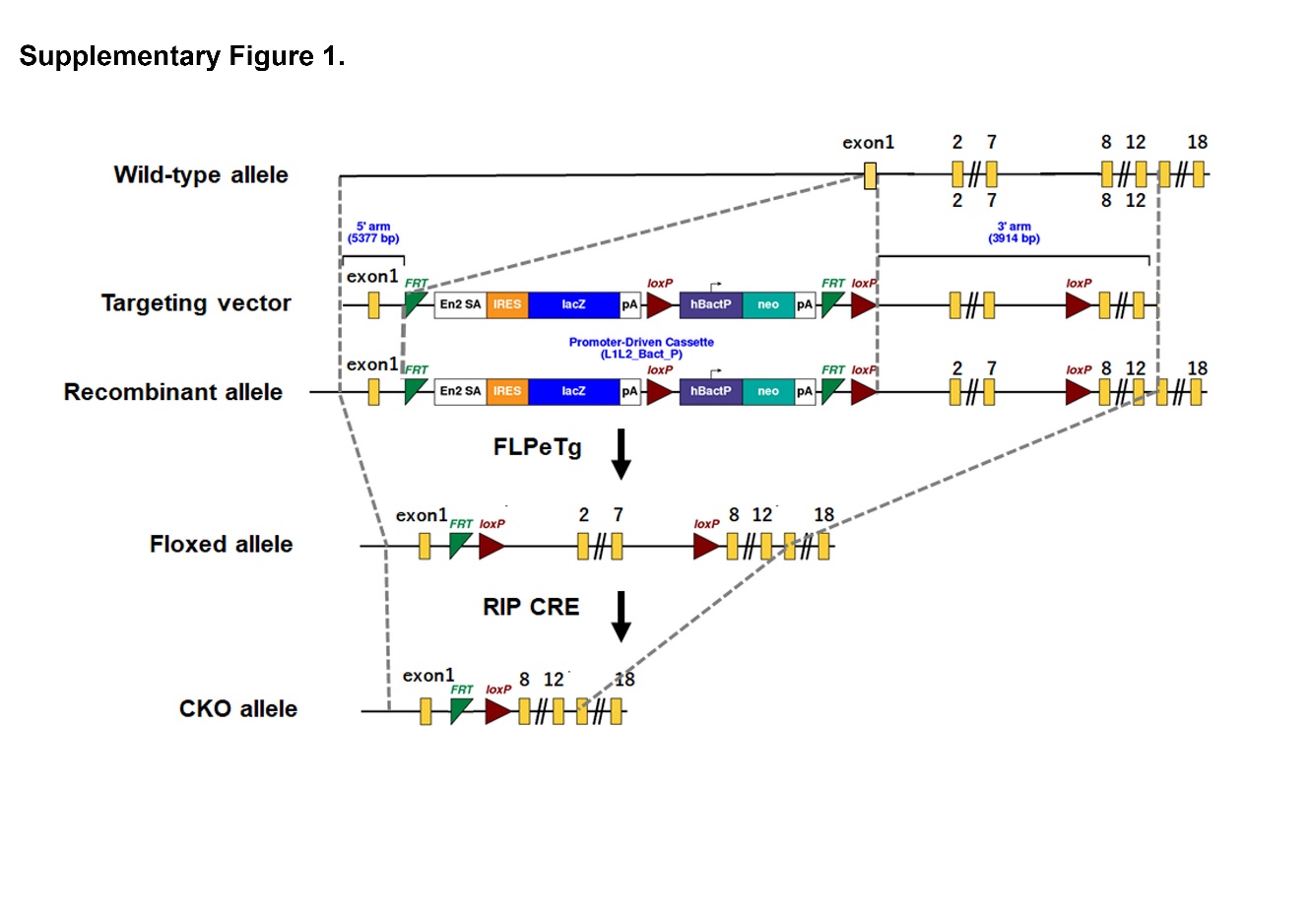


**Supplemental Figure 1.** **Generation of β-cell-specific *Gls2* knockout mice.** The strategy used to generate β-cell-specific *Gls2* knockout mice by homologous recombination. Exon 2-7 of Gls2 sequences were flanked with loxP sites and recombination induced by breeding mice with RIP-Cre transgenics.


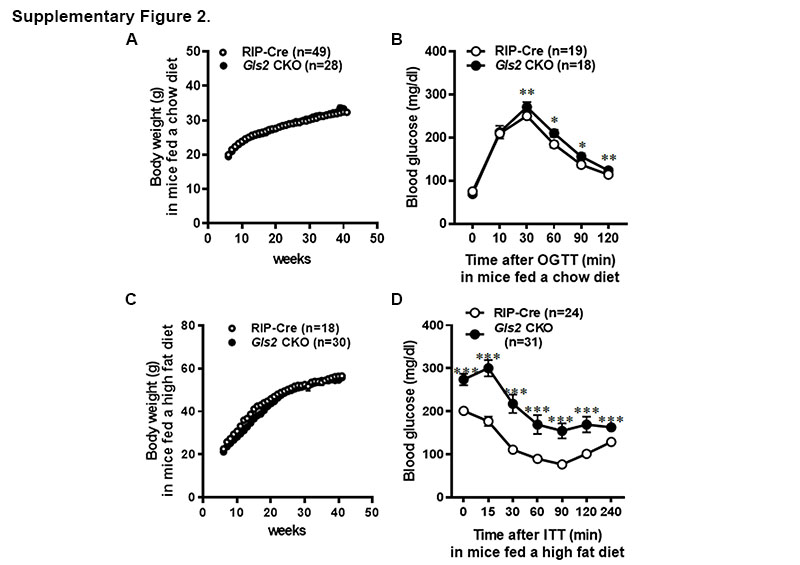


**Supplemental Figure 2.** **Comparison of bodyweight and glucose intolerance in chow diet, and bodyweight and insulin tolerance in high fat diet between β-cell specific *Gls2* conditional knockout mice and RIP-Cre. A-B,** *Gls2* CKO and RIP-Cre mice fed a chow diet were evaluated body weight according to weeks of age in A and performed the oral glucose loading test (OGTT) at 20 weeks of age was shown in B. *Gls2* CKO: β-cell specific *Gls2* conditional knockout mice, and RIP-Cre: control mice. **C,** *Gls2* CKO and RIP-Cre mice fed a high fat diet were evaluated body weight according to weeks of age at 20 weeks of age. D. Insulin tolerance test (ITT) to determine the whole-body sensitivity of insulin receptors by measuring blood glucose level changes before and after insulin administration in *Gls2* CKO and RIP-Cre mice fed a high fat diet. Raw data of the change from baseline are shown. Data are shown in mean ± SEM. *P<0.05 and **P<0.01.


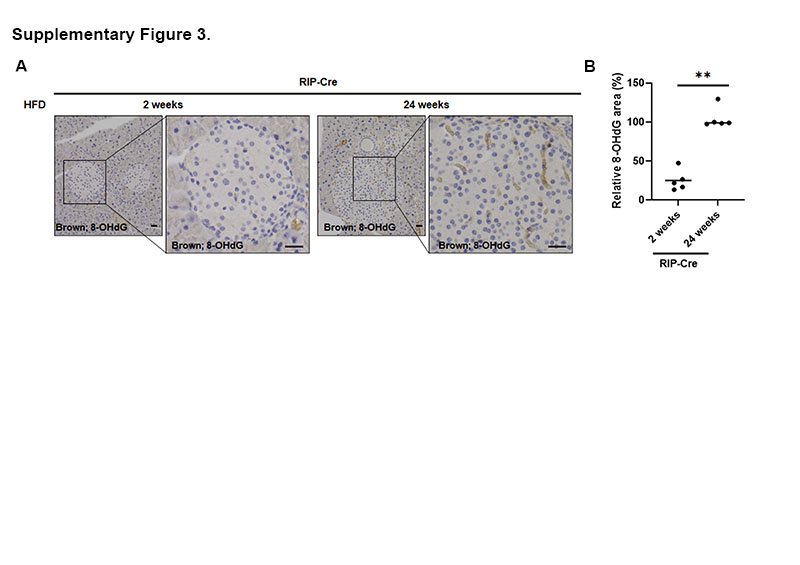


**Supplementary Figure 3. Immunohistochemical analysis of the oxidative stress marker 8-OHdG (shown in brown) in pancreas from RIP-Cre mice fed a HFD for 2 weeks or 24 weeks. A.** Representative images of 8-OHdG. Scale bars: 20 μm. **B.** The relative 8-OHdG area, which was analyzed by Image J.


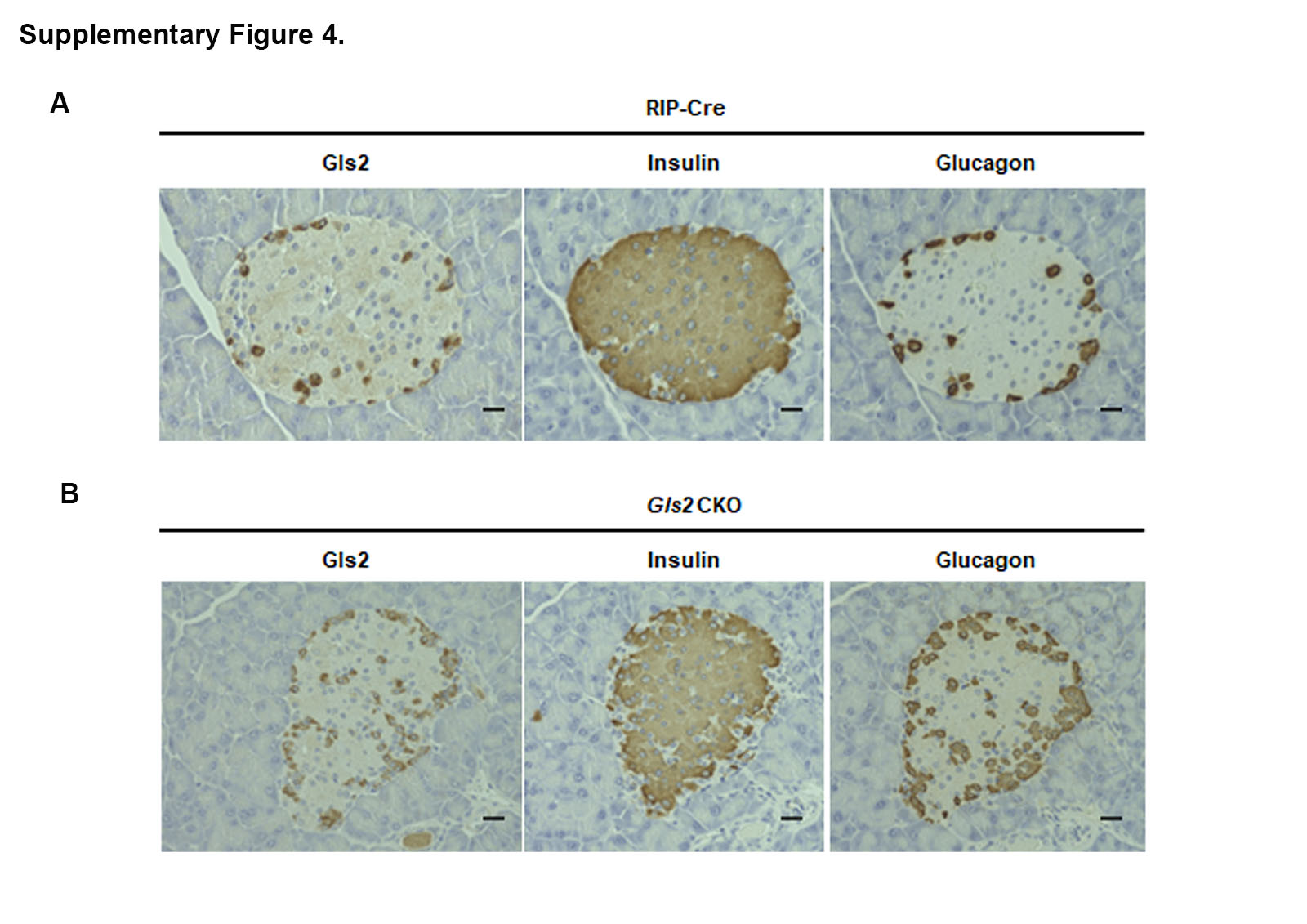


**Supplemental Figure 4.** **The immunohisto-chemical analysis of Gls2 as well as insulin and glucagon in the pancreatic islets for RIP-Cre mice and *Gls2* CKO.** **A-B,** Gls2, Insulin and Glucagon were all shown in brown in RIP-Cre (A) and in *Gls2* CKO (B). Scale bars: 20 μm.


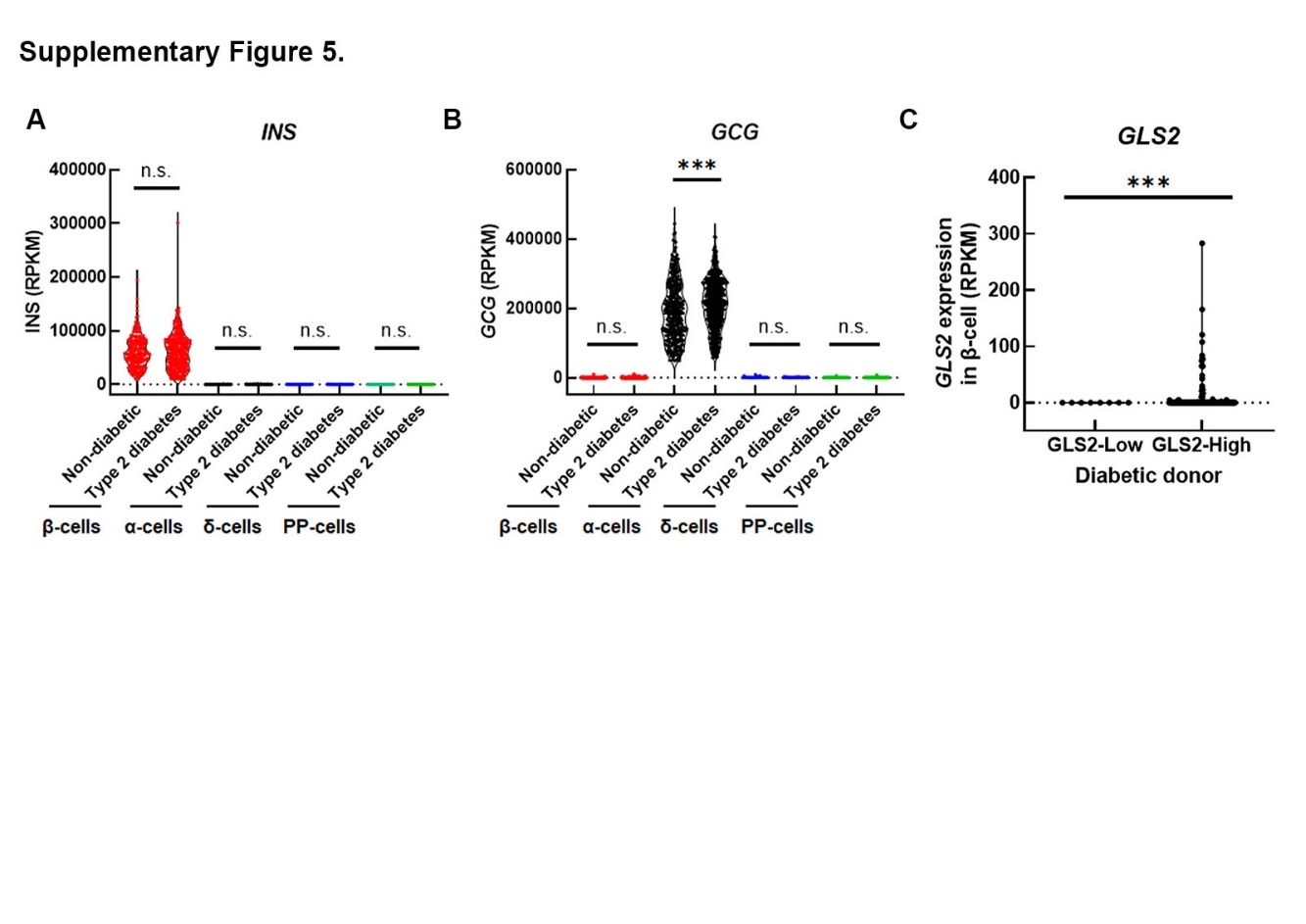


**Supplemental Figure 5.** **Gene expression of *insulin (INS),* *glucagon (GCG)* and *GLS2* using single-cell RNA sequencing data base of human pancreatic islet cells from each type 2 diabetic donor and nondiabetic donors. A-B,** Violin plots of *INS* (A) and *GCG* (B) gene expression in islet cells, including β-cells, α-cells, δ-cells and PP-cells, from type 2 diabetic and nondiabetic donors were assessed using the NCBI database GSE81608. ***P<0.001. n.s., not significant. **C,** Comparison of the violin plots of *GLS2* gene expression between diabetic donors with the lowest *GLS2* expression and diabetic donors with the highest *GLS2* expression. ***P<0.001.
